# Current Applications and Outcomes of Robotic Surgery in Pediatric Upper Airway and Neck Procedures: A Systematic Review

**DOI:** 10.3390/children12060765

**Published:** 2025-06-13

**Authors:** Irene Claudia Visconti, Marella Reale, Virginia Dallari, Eleonora M. C. Trecca, Antonella Miriam Di Lullo, Mario Turri-Zanoni, Michele Gaffuri

**Affiliations:** 1 GOS, Young Otolaryngologists Group of the Italian Society of Otorhinolaryngology–Head and Neck Surgery, 00162 Rome, Italy; ireneclaudia.visconti@auslromagna.it (I.C.V.); marella.reale@meyer.it (M.R.); e.trecca@operapadrepio.it (E.M.C.T.); antonellamiriam.dilullo@unina.it (A.M.D.L.); tzmario@inwind.it (M.T.-Z.); michele.gaffuri@unimi.it (M.G.); 2 Otorhinolaryngology Unit, Morgagni Pierantoni Hospital, Azienda USL della Romagna, 47121 Forlì, Italy; 3 Otorhinolaryngology Unity, Head and Neck Department, Meyer Children’s Hospital IRCCS, 50139 Florence, Italy; 4 Department of Otolaryngology Head and Neck Surgery, Santa Maria delle Croci Hospital, AUSL della Romagna, 48121 Ravenna, Italy; 5 Young Confederation of European ORL-HNS, Y-CEORL-HNS, 1040 Vienna, Austria; 6 Department of Otorhinolaryngology and Maxillofacial Surgery, IRCCS Research Hospital Casa Sollievo della Sofferenza, 71013 San Giovanni Rotondo (FG), Italy; 7 Otorhinolaryngology-Head and Neck Surgery Unit, AORN “San Pio”, 82100 Benevento, Italy; 8 CEINGE-Advanced Biotechnology, 80131 Naples, Italy; 9 Department of Otolaryngology-Head and Neck Surgery, ASST Lariana, Ospedale Sant’Anna, University of Insubria, 22042 Como, Italy; 10 Department of Biotechnology and Life Sciences, University of Insubria, 21100 Varese, Italy; 11 Department of Otolaryngology and Head and Neck Surgery, Fondazione IRCCS Ca’ Granda Ospedale Maggiore Policlinico of Milan, 20122 Milan, Italy; 12 Department of Clinical Sciences and Community Health, University of Milan, 20122 Milan, Italy

**Keywords:** children, Da Vinci, TORS, robotic surgery, OSA, pediatric airway

## Abstract

**Objectives**: This review summarizes current evidence on robotic-assisted upper airway and neck surgery in pediatric patients, highlighting clinical indications, outcomes, limitations, and areas for future research. **Methods**: A systematic review was conducted in accordance with PRISMA guidelines, including studies on robotic surgery for pediatric patients (≤18 years) with upper airway conditions and cervical pathologies. Data on study characteristics, patient demographics, surgical details, outcomes, and robotic system advantages or limitations were extracted. **Results**: Twenty studies met inclusion criteria, comprising 104 pediatric patients who underwent 110 robotic procedures, mostly transoral robotic surgery (TORS) for base of tongue, laryngeal, and cervical pathologies. The Da Vinci Si was the most used system. The mean operative time was ~74 min, with minimal blood loss and no intra/post operative tracheostomies. Reported advantages included enhanced visualization, precision, and reduced morbidity. Limitations involved size mismatches, limited working space, and high costs. Follow-up (mean 11.4 months) revealed no recurrences, confirming feasibility and safety in selected pediatric cases. **Conclusions**: Robotic-assisted surgery appears to be a feasible and safe option for managing pediatric upper airway and neck conditions, offering promising functional and aesthetic outcomes with low complication rates. However, its use is currently limited by anatomical constraints, high costs, and the need for surgeon training. Long-term prospective studies with larger cohorts are needed to confirm its efficacy and define its role compared to traditional techniques.

## 1. Introduction

Transoral robotic surgery (TORS) has become an established technique in the treatment of oropharyngeal, hypopharyngeal, and laryngeal pathologies in adults, with numerous studies demonstrating its feasibility and efficacy in this population [1,2]. In contrast, the adoption of robotic-assisted procedures in pediatric otolaryngology remains relatively limited, although robotic surgery has already gained traction in pediatric abdominal, thoracic, urological, and gynecological procedures due to its precision and minimally invasive nature [3]. The first reported application of TORS in children was in 2007, when Rahbar et al. [4] described the use of robotic assistance for laryngeal cleft repair in five pediatric patients. However, the study highlighted the limitations of the robotic platform at that time, particularly regarding the size of the robotic arms and limited surgical exposure, which prevented completion in three of the five cases due to inadequate visualization and restricted instrument [4]. Since then, technological advancements, including improved optics, miniaturization of instruments, and better patient selection, have contributed to the gradual increase in reported pediatric TORS procedures.

Several case reports and small case series have demonstrated the feasibility of robotic-assisted approaches in pediatric head and neck surgery, including sleep apnea interventions, airway reconstructions, and excision of pharyngeal masses [5,6,7]. Notably, the literature also includes rare cases of robot-assisted procedures for pediatric neck masses using alternative approaches, such as transhairline incisions [8] or retroauricular access [9], which allow mass removal through small, well-concealed skin incisions that minimize visible scarring. These minimally invasive approaches are gaining popularity for their favorable surgical, functional, and cosmetic outcomes.

Recent evidence has emphasized the importance of careful patient selection, preoperative planning, and a multidisciplinary approach to ensure safety and optimize outcomes in children [10]. Furthermore, emerging data suggest that the integration of robotic techniques may reduce surgical morbidity, hospital stay, and recovery time, while improving functional outcomes, particularly in complex airway procedures [11].

Nonetheless, the literature remains sparse, with most publications describing isolated experiences rather than large-scale or comparative studies. The pediatric application of robotic surgery faces important challenges: the bulky system, high costs, steep learning curve, and need for adaptation to smaller, delicate anatomy remain significant barriers [10,12]. As Johnston et al. [12] suggest, the potential of robotic surgery to expand pediatric otolaryngology indications depends on ongoing technological improvements and the development of pediatric-specific training.

This systematic review aims to summarize current evidence on the use of robotic-assisted surgery for upper airway and neck procedures in pediatric patients, with a focus on clinical indications, surgical outcomes, and limitations, while also identifying areas that require further investigation.

## 2. Materials and Methods

After registering with the PROSPERO database (ID CRD420251059669), this systematic review was conducted in accordance with the Preferred Reporting Items for Systematic Reviews and Meta-Analyses (PRISMA) guidelines [13].

A comprehensive literature search was performed using PubMed, Scopus, and Web of Science databases, employing the following search string: “robotic surgery” [All Fields] AND (“TORS” [All Fields] OR “otorhinolaryngology” [All Fields] OR “upper airways” [All Fields] OR “adenotonsillar hypertrophy” [All Fields] OR “tonsillar hypertrophy” [All Fields] OR “laryngology” [All Fields] OR “pediatrics” [All Fields] OR “children” [All Fields]). Reference lists of the included studies were also manually screened to identify additional relevant articles. All titles and abstracts published up to 23 April 2025 were independently reviewed by two authors (I.C.V. and M.R.). Disagreements regarding study inclusion were resolved through discussion. 

The inclusion criteria were original articles and case reports written in English, describing pediatric patients (aged ≤ 18 years) with congenital or acquired upper airway and neck pathologies—either benign or malignant—who underwent robotic surgery, with an available abstract.

Exclusion criteria comprised non-original publications (e.g., reviews, meta-analyses, editorials, letters to the editor, consensus statements, conference abstracts, “how I do it” articles), studies not related to robotic surgery, or those involving mixed populations of pediatric and adult patients. Also, cases in which the robotic approach failed and it was converted were excluded from the final analysis.

Full texts of the eligible studies were reviewed, and their reference lists were examined to identify additional eligible articles. The extracted data included general study characteristics, demographic and clinical information about the pediatric patients, the type of surgical procedure, robotic system used, complications, follow-up duration, recurrence, and the reported advantages or limitations of the robotic approach.

## 3. Results

**Figure 1:**
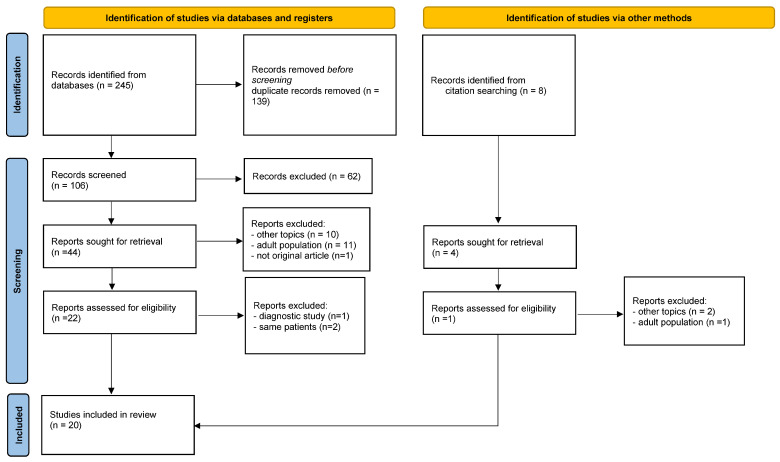
Prisma flow diagram of the review [13].

The study selection process is illustrated in the PRISMA flow diagram (Figure 1).

A total of 20 studies met the inclusion criteria: 10 case reports, 5 retrospective studies, 4 case series, and 1 prospective study. In total, 104 pediatric patients underwent 110 robotic procedures (Table 1). Geographically, 11 studies originated from the United States, 6 from Europe (Turkey, Italy, and France), and 3 from Asia (India and China). The study with the largest sample size was conducted by Worden et al. [14] encompassing 40 patients.

The most common pathological site was the base of the tongue (60/110, 54.55%), followed by the larynx (26/110, 23.64%), the cervical region (22/110, 20.00%), and the oropharynx (2/110, 1.82%).

Table 2 summarized the treated conditions. Accordingly, the predominant surgical approach was TORS for lesion excision and/or tissue reduction (75/110 cases; 68.2%), followed by cleft repair (21/110; 19.1%), release procedures of aerodigestive tract strictures (6/110; 5.5%), retroauricular excision (6/110; 5.5%), robot-assisted hyoepiglottopexy (1/110; 0.9%), and posterior cordectomy with subtotal arytenoidectomy (1/110; 0.9%).

The associated procedures described included neoadjuvant and adjuvant chemotherapy and radiotherapy in malignant cases, with one case also requiring a neck dissection performed via cervicotomy. In another instance, CO_2_ laser was utilized through a robotic arm. Additionally, two cases required a combined external approach to achieve complete excision of the pathology. 

The mean operative time, reported for 84 procedures, was 74.48 ± 72.69 min. For the 35 procedures where docking time was available, the mean docking time was 14.95 ± 10.05 min. Blood loss data were reported in 31 procedures, in which 1 case involved <5 mL, 27 cases between 5 and 10 mL, 2 cases between 10 and 15 mL, and 1 case with 250 mL due to external jugular vein rupture. The most frequently utilized robotic platform was the Da Vinci Si system (91/110 procedures, 82.73%), followed by the Da Vinci Xi system (13/110, 11.82%).

Preoperative tracheostomy was present in 8 out of 104 patients (7.69%); no intraoperative or postoperative tracheostomies were required.

Hospitalization data were available for 72 procedures, with a mean length of stay of 5.11 ± 5.66 days. Two studies reported hospital stays ranging from 1 to 14 days (Table 3).

The most frequently cited advantages across studies included high-resolution three-dimensional visualization, tremor filtration, and enhanced instrument maneuverability. Additional reported benefits included high magnification, minimally invasive access, fewer postoperative complications, shorter hospital stays, improved swallowing function, and lower morbidity. Overall, robotic surgery was consistently described as a feasible and safe alternative to other transoral approaches.

Nevertheless, several limitations were noted. Chief among them were the restricted operative field and the fact that current robotic systems are not specifically designed for pediatric anatomy, leading to size mismatches between the robotic arms and the oral cavity. Other drawbacks included high costs, lengthy setup and docking times, and potential robotic arm collisions that can disrupt intraoperative workflow. Limited visualization and challenges in assisting bedside surgeons were also mentioned in some cases.

The mean follow-up duration among the 18 studies with available data was approximately 11.4 months. No recurrences were observed during the follow-up period, although one laryngeal neurofibroma required retreatment for residual disease. One patient remained tracheostomy-dependent at 12 months, and another required a second transoral laser microsurgery (TLM) procedure.

## 4. Discussion

TORS is a widely accepted approach for the management of both benign and malignant otolaryngologic conditions in adults [27,28]. Due to its several advantages, its use has increasingly extended to pediatric airway pathologies in recent years [14]. This systematic review investigates the indications, safety profile, clinical outcomes, and potential advantages and limitations of robotic-assisted surgical techniques in the management of upper airway and neck pathologies in the pediatric population.

The first work on this topic by Rahbar et al. [4] reports the use of TORS for the repair of laryngeal clefts. According to the authors, robotic-assisted surgery was feasible and effective, though only in highly selected cases. In fact, only 21 patients with laryngeal cleft had been treated using robotic technology up to that point [4,5,14]. Although enhanced visualization and instrument articulation improve maneuvers to manage complex airway anomalies in confined anatomical spaces, limited transoral access requires careful patient selection and technological advancements to enhance applicability [4,11,27]. Over the years, the number of reported cases has increased, while most of the studies included in the review are still case reports. In 2024, Worden et al. [14] presented the largest case series to date, involving 40 patients and demonstrating that TORS outcomes—including operative times, complication rates, and hospital stays—were comparable to traditional surgical methods. In case of type I laryngeal clefts and lymphatic malformations, operative times were generally longer with the robotic approach. However, postoperative swallow outcomes were significantly improved in patients undergoing TORS for type I laryngeal cleft repair. In this regard, robotic-assisted surgery demonstrated promising functional results in management of pediatric palatal clefts, including improvements on middle ear function and hearing. In these patients, shorter hospital stays and a reduced incidence of otitis media with effusion (OME) were noted [29,30,31,32].

According to our findings, the main field of application of robotic surgery in the pediatric population is represented by the treatment of obstructive sleep apnea (OSA) which is often erroneously attributed solely to adenotonsillar hypertrophy. Although adenotonsillectomy represents the first-line treatment for pediatric OSA [33], up to 40% of children may have persistent OSA despite the surgical procedure [18]. Base of tongue and lingual tonsil hypertrophy are well-known contributors to the residual airway obstruction [18,33]. In the literature, many papers have highlighted the efficacy of TORS in reducing the severity of OSA [7,19], improving visualization and minimizing morbidity, when compared to open procedures [5,34,35]. Leonardis et al. [18] reported positive and consistent results with an acceptable length of hospital stay and low rate of postoperative complications. Similarly, Thottam et al. [19] concluded that TORS may be a suitable and safe option for children with residual OSA after adenotonsillectomy and low compliance to other medical therapy. Montevecchi et al. [6] also emphasized the importance of careful patient selection for successful TORS application.

In recent years, the treatment of lingual thyroglossal duct cysts (LTGDC) has become an emerging application of pediatric robotic surgery. Although rare, these cysts may lead to dysphagia, airway obstruction, and OSA [36]. While some authors advocate for simple marsupialization or excision, high recurrence rates have also been reported [37,38]. Johnston et al. [12] highlighted the advantages of TORS in safely accessing and excising cysts located in the post-hyoid space. Similar positive outcomes were observed in studies by Kayhan et al. [7,17] and Turhan et al. [39].

Robotic approaches have also shown value in the excision of cervical masses, offering high surgical precision and improved cosmetic results, particularly when avoiding visible scars is a priority [8,39]. In this review, Lin et al. [8] and Venkatakarthikeyan et al. [9] report cases of cervical mass excision performed through a retroauricolar/transhairline approach highlighting the possibility of complete removal with acceptable aesthetic results, shorter hospital stays, and fewer complications. Moreover, the use of the Da Vinci Xi system in four out of six patients, as reported by the authors, is likely attributable to its slimmer robotic arms, improved maneuverability in confined anatomical spaces, and more efficient docking process—all features frequently emphasized in the literature and particularly advantageous for pediatric head and neck procedures [40,41].

Furthermore, the challenges of accessing and visualizing the narrow spaces of the pediatric airway leads to the increasing adoption of advanced technologies; not only robotic surgery but also the exoscope has been considered. Gaffuri et al. [42] propose that high-definition 3D 4K exoscopy may offer a valuable alternative, helping to overcome the visual limitations of traditional surgical approaches.

Airway management during robotic surgery remains a debated issue, especially in pediatric patients. Although orotracheal or nasotracheal intubation could make the management of small airways challenging, only eight tracheostomies were reported in the included studies, all of which were performed preoperatively [7,15,18,21,23]. As highlighted by Leonardis et al. [18], appropriate tube selection and placement did not interfere with instrument mobility during surgery.

The feasibility of robotic surgery is further supported by an analysis of hospitalization length and complications. According to our findings, the length of stay ranges from a minimum of one day to a maximum of 23 with a mean of 5.11 ± 5.66 days. Given the delicate surgical site and the young patient population, the observed hospitalization duration appears consistent with expectations. Similar results have been described in terms of complications. Minor bleeding has been reported in some cases [7,12,18,19], which was more likely attributable to the high vascularity of the surgical site rather than the robotic technology itself. On the other hand, when considering the need for additional surgery, four cases of revision surgery have been reported by Arnold et al. [23] and Worden et al. [14]. The authors attributed these revisions primarily to the size of the robotic instruments and the limited visualization and accessibility of the surgical field.

Despite these encouraging findings, several limitations have been acknowledged in the literature.

First, a major constraint is that the Da Vinci Surgical System was not specifically designed for pediatric patients or for the narrow anatomical confines of the upper airway. The large size of the instruments limits their maneuverability, prompting calls for the development of smaller, pediatric-specific robotic tools. Hockstein et al. [41] similarly advocated for the miniaturization of robotic components to better accommodate pediatric applications.

In addition, novice surgeons often experience longer operative times and higher complication rates during their initial cases. This highlights the importance of dedicated training programs tailored to pediatric robotic surgery. Structured curricula incorporating simulation-based learning, mentorship, and supervised operating room experience are strongly recommended [43,44].

Another significant barrier is the high cost of robotic systems. Although reduced hospital stays and fewer complications may help to offset these expenses, comprehensive economic evaluations are needed to determine the true cost-effectiveness of these procedures [45,46].

In conclusion, despite growing interest and expanding clinical experience, high-quality evidence in the field of pediatric head and neck robotic surgery remains limited, underscoring the need for further well-designed studies to validate its safety, efficacy, and long-term outcomes. As reported by Gottman et al. [11], most existing studies are retrospective in nature, with small sample sizes and moderate heterogeneity, which may limit the reliability of their findings. Future research should prioritize prospective, multicenter randomized controlled trials involving larger cohorts. In particular, long-term follow-up data are needed to assess the role of TORS in the management of residual OSA following adenotonsillectomy. The development of specific guidelines for pediatric robotic surgery through future prospective and standardized studies would be desirable. This could promote broader clinical adoption and support the establishment of highly specialized pediatric centers.

## 5. Conclusions

This review highlights that robotic-assisted surgery may be considered a feasible and safe approach for managing pediatric upper airway procedures, with favorable outcomes in terms of operative times, complication rates, and hospitalization. It offers additional benefits such as improved functional outcomes, low complication rates, and superior cosmetic results. However, long-term prospective studies with larger patient cohorts are strongly needed to objectively assess its efficacy and to enable more robust comparisons with other surgical techniques.

## Figures and Tables

**Table 1:** Overview of the included studies and robotic procedures in pediatric airway and neck surgery. BOT, base of tongue; OSA, obstructive sleep apnea; LTGDC, lingual thyroglossal duct cyst; TORS, transoral robotic surgery; TBR, tongue base reduction; TLM, transoral laser microsurgery; FAMM, facial artery musculomucosal flap; SD, standard deviation; n.a., not available.

	Type of Study	Patients (*n*)	Mean Age (*n* ± SD) Years	Pathology	Localization	Procedure	Docking Time	Surgery Time	Da Vinci Si	Da Vinci Xi	Tracheotomy	Hospitalization Days	Complications
Rahbar (2007) [4]	Retrospective	2	5	Laryngeal cleft (2)	Larynx	Laryngeal cleft repair (2)	n.a.	n.a.	2	0	0	n.a.	n.a.
Kokot (2013) [15]	Case Report	1	15	Oropharyngeal synovial sarcoma	Oropharyngeal wall	Partial pharyngectomy and partial glossectomy	n.a.	n.a.	1	0	1	3	0
Wine (2013) [16]	Case Report	1	1, 5	High grade undifferentiated sarcoma	Soft palate (oropharynx)	Resection of the left hemipalate and lateral oropharynx, partial base of tongue resection + FAMM	n.a.	226 min	1	0	0	16	0
Kayhan (2013) [17]	Case Report	1	0, 2	LTGDC	BOT	TBR	n.a.	3 min	1	0	0	3	0
Leonardis (2013) [18]	Retrospective study	16	12	OSA (11), disphagia (2), upper airway obstruction (1), upper airway obstruction due to exercise (1), lingual tonsillitis (1)	BOT	TORS	6 min	34 min	16	0	2	1–13	Minor bleeding (2), pneumonia (2), fever of unclear etiology (1), poor pain control (4)
Ferrell (2014) [5]	Case series	3	6	Posterior glottic stenosis (1), laryngeal cleft (1), idiopathic bilateral vocal crod paralysis (1)	Larynx	Posterior cricoid spli with cartilage graft placement (1), simple repair (1), posterior cordectom + subtotal arytenoidectomy (1) Conversion needed (3)	n.a.	n.a.	3	n.a.	n.a.	0	Oral tongue edema, decreased oral intake, and suspected aspiration (5 days later) (1)
Thottam (2015) [19]	Retrospective study	9	10, 5	OSA	BOT	TORS	n.a.	n.a.	9	0	0	1–14 dqys	Bleeding (1), pneumonia (1)
Carroll (2016) [20]	Case Report	1	6	LTGDC	BOT	Resection of tongue muscle with the mass. No hyoid bone resection	15 min	28 min	1	0	0	1	0
Colaianni (2017) [21]	Case report	1	9	Epiglottic prolapse	Larynx	Robot-assisted Hyoepyglottopexy	n.a.	n.a.	1	0	1	n.a.	0
Montevecchi (2017) [6]	Case series	3	13, 25	OSA	BOT	TORS (1), TORS + epiglottoplaty (1), TBR + epiglottoplasty + adenotonsillectomy (1)	15 min	60 min	3	0	0	4, 66	0
Canevari (2017) [22]	Case report	1	16	Ewing Sarcoma	BOT	Partial glossectomy TORS	n.a.	n.a.	1	0	0	2	0
Kayhan (2017) [7]	Case series	8	5, 1	LTGDC (4), vallecular cyst (1), lingual thyroid (1), minor salivqry gland tumor (1), bronchogenic cyst (1)	BOT	TORS	6 min	8.8 ± 6.9 min	7	1	1	2, 25 ± 1.4	Minor bleeding 10 days after surgery (1)
Arnold (2018) [23]	Case report	1	6	neurofibroma	Supraglottic extending laterally into the parapharyngeal and carotid space	TORS	40 min	50 min	0	1	1	23	TLM laser CO_2_ for residual mass in left false vocal fold
Turhan (2019) [24]	Case report	1	0, 4	LTGDC	BOT	excision	n.a.	10 min	0	1	0	7	0
Fanous A (2020) [25]	Case report	1	6	Branchial cyst I arch	Parapharyngeal space	TORS	n.a.	n.a.	0	1	0	4	0
Venkatakarthikeyan (2020) [9]	Case series	3	10, 66	Dermoid cyst of BOT, Branchial cyst (II arch), acinic cell carcinoma	BOT, neck region, left parotid	Removal of the mass	12, 33 min	n.a.	0	3	0	2, 5	0
Lin HJ (2021) [8]	Prospective longitudinal cohort study	4	4.4	Congenital cervical lymphatic malformations	Neck region	Trans-hairline approach	5.5	106 min	2	2	0	n.a.	Intraoperative injury to the external jugular vein (1)
Johnston (2023) [12]	Case series	7	8	LTGDC (3 primary, 4 recurrent)	BOT	Removal of the mass + hyoid bone (4), removal of the mass (hyoid bone previously removed, 3)	n.a.	n.a.	0	7	0	2, 3	External fistula (1), minor bleeding
Das (2023) [26]	Case report	1	7	LTGDC	BOT	TORS	n.a.	10min	0	1	0	7	0
Worden CP (2024) [14]	Retrospective study	40	6, 41	Laryngeal cleft (18), Lynphatic malformation (9), BOT mass (7), bilateral fold paralysis/posterior glottid stenosis (2), Aerodigestive Tract Stricture (6), Saccular Cyst/Neurofibroma (4)	Larynx, BOT	Removal of mass, correction of laryngeal cleft, release, TORS	n.a.	145 min	40	n.a.	n.a.	5.65	Surgical failure/Required revision surgery (3), Granulation tissue/Granuloma (2), Post-extubation laryngospasm/bronchospasm (1) Aerodigestive tract stricture (1) Pneumonia (1)

**Table 2:** Summary of conditions treated with robotic-assisted surgery in the included pediatric cases. OSA, obstructive sleep apnea; LTGDC, lingual thyroglossal duct cyst; BOT, base of tongue.

Condition Treated	*n* (%)
OSA-lingual tonsillar hypertrophy	25 (22.7%)
Laryngeal Cleft	21 (19.1%)
LTGDC	15 (13.6%)
BOT masses	7 (6.3%)
Cervical lymphatic malformations	13 (11.8%)
Malignant tumor *	5 (4.5%)
Cystic lesions **	10 (9.0%)
Bilateral vocal fold paralysis or posterior glottic stenosis	3 (2.7%)
Lingual thyroid	1 (0.9%)
Aerodigestive tract strictures	6 (5.5%)
Epiglottic prolapse	1 (0.9%)
Exercise-induced respiratory difficulty	1 (0.9%)
Dysphagia	2 (1.8%)

* Malignant tumor: Acinic cell carcinoma (left parotid), pleomorphic adenoma (minor salivary glands), extraskeletal Ewing’s sarcoma (tongue), high-grade undifferentiated sarcoma (soft palate), and oropharyngeal synovial sarcoma (oropharyngeal wall). ** Cystic lesions: Five neurofibromas or laryngeal saccular cysts, one first branchial cleft cyst, one second branchial cleft cyst, one dermoid cyst, one vallecular cyst, one bronchogenic cyst.

**Table 3:** Summary of study characteristics, patient demographics, procedural data, and complications.

Data	Results
***Retrospective studies***	10 (50%)
***Case Report***	5 (25%)
***Case Series***	4 (20%)
***Prospective study***	1 (5%)
***Patients (n)***	104
***Procedures (n)***	110
***Mean Age***	7.9 years
***Da Vinci Si***	91 (82, 73%)
***Da Vinci Xi***	13 (11, 82%)
***Tracheostomy***	8 (7.69%)
***Hospitalization***	5.11 (± 5.66)
Complications *	28 (25.4%)

* Complications: In total, 5 minor bleedings, 4 pneumonias, 4 inadequate pain controls, 3 surgical failure/revision surgeries required, 2 granulation tissues or granuloma formations, 1 aerodigestive tract stricture, 1 fever of unknown origin, 1 intraoperative injury to the external jugular vein, 1 oral tongue edema, decreased oral intake, and suspected aspiration, 1 pharyngocutaneous fistula, and 1 post-extubation laryngospasm/bronchospasm.

## Abbreviations

The following abbreviations are used in this manuscript:

TORS	Transoral Robotic Surgery
PRISMA	Preferred Reporting Items for Systematic Reviews and Meta-Analyses
BOT	Base Of Tongue
OSA	Obstructive Sleep Apnea
LTGDC	Lingual Thyroglossal Duct Cyst
TBR	Tongue Base Reduction
TLM	Transoral Laser Microsurgery
FAMM	Facial Artery Musculomucosal Flap
SD	Standard Deviation
n.a.	Not Available
